# Perceptions of Own Illness among the Elderly as Measured by the Brief-IPQ Scale and the IPIS

**DOI:** 10.3390/ijerph19084665

**Published:** 2022-04-12

**Authors:** Katarzyna Pawlikowska-Łagód, Magdalena Suchodolska

**Affiliations:** 1 Department of Humanities and Social Medicine, Faculty of Medical Sciences, Medical University of Lublin, 20-059 Lublin, Poland; 2 Faculty of Medicine, Medical University of Lublin, 20-059 Lublin, Poland; magda.suchodolska.1998@interia.eu

**Keywords:** perceptions of own illness, the elderly, illness, chronic illness

## Abstract

Background: The perception of one’s own illness by the elderly significantly affects the success of diagnostic and therapeutic processes. It depends on the patient’s existential situation, coping strategies, social support and the way the patient is treated by the medical personnel. The aim of this study was to investigate how the elderly perceive their own illness. Methods: The study covered 303 people over 60 years of age who were hospitalized in wards of Lublin hospitals. A diagnostic survey method was used in the study. The research tools were: the Illness Perception Questionnaire (Brief-IPQ), Imagination and Perception of Illness Scale (IPIS) and a questionnaire of own authorship. The place of residence in the study population significantly influenced the perception of own illness measured by the Brief-IPQ. Results: The elderly perceived their own illness through the prism of mental and physical destruction. Statistically significant correlations were observed between almost all components of the Brief-IPQ and self-assessed health status of the subjects. Conclusions: Older people’s perception of their own illness is very important in the success of the therapeutic process. The perception of disease is not statistical; it changes depending on the chronicity of the disease, its duration, cultural factors and the doctor–patient relationship. It is associated with biopsychosocial processes that affect the ability to perceive and understand the disease and to take appropriate actions to improve health.

## 1. Introduction

The progressive aging of society is currently one of the main problems of modern civilization. The development of this phenomenon is caused by the extension and improvement of the quality of human life, negative demographic growth as well as civilization and technological progress. The increasingly rapid population aging has an impact on the demographic, economic and social sphere of the state and on health care policy. As a result, this process poses many challenges for health care representatives in terms of medical services aimed at the elderly, as well as their availability, ensuring professional and dignified treatment and creating conditions for adequate prevention and health education [1,2,3].

The elderly patients constitute a characteristic patient, as they are accompanied by multimorbidity. For this reason, there is a need for the senior citizen to receive treatment from several specialists in different medical institutions. A factor that can significantly affect cooperation with the physician and thus the therapeutic processes is the patient’s perception of their own illness [1]. A person convinced of the possibility of recovery and control over the disease is more inclined to good cooperation with the medical personnel. In chronically ill patients, a positive perception of their own illness also influences a better quality of life, while patients who have a negative perception of their own illness may develop other negative health disorders [2,4].

Perception of own illness is a concept that is derived from self-regulation theory, which describes people as active problem solvers. Furthermore, this theory attempts to explain how individuals behave by recognizing, coping with and evaluating steps to reduce the perceived gap between current and ideal health. Thus, the perception of own illness is a self-declared belief about one’s own health [5]. Another definition specifies that illness perception is the patients’ organized beliefs relating to the characteristics of their illness. Leventhal (1984) identified five basic dimensions relating to perception of own illness:

1. Beliefs about common symptoms of disease—identity;
2. Perceived duration of illness—timeline;
3. Causal factors;
4. Expected effects and outcome of the disease—consequence;
5. Possibility of recovery and controllability of the disease [6].

It should be noted that the causal dimension of a chronically ill person’s perception of own illness is the patient’s individual opinion as to what may have caused the illness. However, this dimension depends on many important factors. It is based on, among others, personal experiences of the patient, conversations and opinions of loved ones, attitudes of health care professionals and created information in the media relating to the disease [7].

So far, only a few studies on the perception of own disease have been carried out, measured using the Disease Perception-Brief-IPQ Questionnaire and Imagination and Perception of Illness Scale—IPIS (IPIS) only in the group of elderly people. The use of these two research tools to determine how the elderly perceive their own disease may contribute to broadening the knowledge on the psychosocial aspects of the functioning of the elderly in the situation of the disease [1].

## 2. Materials and Methods

The aim of this study was to investigate how the elderly perceive their own illness. The study involved 303 people over 60 years of age who were hospitalized in the wards of Lublin hospitals: Department of Internal Diseases, Department of Cardiology with Heart Catheter Laboratory, Electrotherapy Laboratory, Echocardiography Laboratory, Rehabilitation Department of the 1st Military Clinical Hospital in Lublin and the Department of Gastroenterology with Endoscopy Laboratory of the Independent Public Clinical Hospital No. 4 in Lublin. A non-randomized purposive scheme was used to select the population. The study was conducted between June 2018 and January 2019.

The following inclusion criteria were adopted for the study group:

- Subjects in the age group of 60 years and above;
- Subjects hospitalized for a specific disease at least once in the 12 months prior to the study;
- Subjects giving informed consent to participate in the study.

Criteria for exclusion from the study group:

- Persons under 60 years of age;
- People declaring no chronic diseases in them;
- Persons who do not consent to participate in the study.

The implementation of the research procedure began after obtaining a positive opinion of the Bioethics Committee at the Medical University of Lublin (KE-0254/109/2016).

A diagnostic survey method was used in the study, and the research tools were: the Illness Perception Questionnaire Brief-IPQ, Imagination and Perception of Illness Scale (IPIS) and a questionnaire of own authorship. The research technique used to collect the research material was direct questioning.

Two research tools examining the perception of one’s own illness were used in the work. Due to the authors’ willingness to investigate the broadest possible spectrum of perception of their own disease in the elderly, not one, but two questionnaires were used. Both the Brief-IPQ and the IPIS focus on the cognitive and emotional dimensions of the perception of the disease, which were important to the authors of this study.

The IPIS questionnaire prepared by J. Sak refers to the semantic differential technique. It consists of 32 contrasting terms concerning the perception of illness. In previous studies, the following components of the IPIS were distinguished: loss of motivation (8 items), destruction of mental sphere (6 items), destruction of physical sphere (8 items), pessimism (4 items), threat to the environment (3 items) and loss of control over the disease (3 items). The tool can be used both among sick people in relation to their subjective perception of illness, and healthy people to investigate the perceptions of individual disease entities. The respondents put their answers on a seven-point scale, where “0” means the selection of the maximum positive trait attributed to the disease, and digit “6” means the selection of the maximum negative trait. Having a high score on a particular IPIS dimension means a more negative perception of disease. The reliability of the above scale was determined using the Cronbach’s α coefficient, which exceeded the value of 0.7. The tool can be used both among sick and healthy people [8].

The Illness Perception Questionnaire Brief-IPQ was created by Broadbent, Petrie, Main and Weinman (2006). The main purpose of this questionnaire is to assess the cognitive and emotional perception of illness. The shortened version of this tool consists of 8 closed questions and one open-ended question [9]. The first 5 assess the cognitive perception of 46 such as impact on life (item 1), duration of illness (item 2), control of illness (item 3), confidence in treatment (item 4) and experience of symptoms (item 5). Items 6 and 8 assess emotional aspects, including concerns about the disease and a multifaceted question regarding their mood. Item 7 assesses the degree of understanding of illness [10].

The respondents place the answers to these questions on the numeric rating scale from 0 (minimum) to 10 points (maximum). The authors of the questionnaire assessed its reliability with the use of the retest test among 132 outpatients. The test repetition has been shown to be very reliable (Pearson correlations 0.24–0.73). The equivalent scales of short IPQ and IPQ-R had moderate to good correlations when tested for simultaneity (Pearson correlations 0.32–0.63). The discriminant validity of the questionnaire was confirmed by its ability to distinguish various diseases, namely, asthma, diabetes, colds, myocardial infarction before discharge from the hospital and the predisposition of patients with chest pain awaiting exercise tests [11]. The obtained results were presented using the arithmetic mean. The Polish version of the questionnaire was adapted by Kossakowska M., who agreed to use the above-mentioned tools in her own research. In her research, she assessed the psychometric value of the Polish version of the questionnaire. The results it obtained confirm its usefulness, despite some deviations from the original version [12].

The author’s own survey questionnaire included questions on the sociodemographic aspects of the respondents. Research on the age, sex, place of residence, education, marital status and self-assessment of the health condition of the respondents was included. The respondents also specified the disease for which they were hospitalized. The questions are disjunctive, i.e., they allow for only one correct answer. Correct answers are coded as 1, while incorrect answers as 0. The research was voluntary, individual, anonymous and realized in accordance with the assumptions of the Helsinki Declaration.

## 3. Statistical Analysis Methods Used

After analyzing the distribution of the controlled dependent variables, it was found that these distributions differed statistically significantly from normal distributions. As a result, non-parametric tests were used to analyze the obtained data. The Mann–Whitney U test was used for comparisons between the two groups. Comparisons between more than two groups were made using the Kruskal–Wallis ANOVA test. The correlations between the controlled variables expressed in quantitative scales were investigated using the Spearman’s rank correlation coefficient—rho. The range of statistical significance was *p* < 0.05, and *p* values between 0.05 and 0.01 were interpreted as statistical trends. All statistical analyses were performed using Statistica, version 12.5.192.0 PL (StatSoft Inc., Kraków, Poland).

## 4. Results

The research results show that almost half of the respondents (45.21%) assessed their health as average. Only 26.07% assessed their health as good, while 0.66% said very good. Diabetes mellitus (12.54%), ischemic heart disease (11.22%) and myocardial infarction (9.90%) were the most common diseases among the study group. Table 1 presents the socio-demographic data of the studied group (sex, place of residence, marital status, level of education) (Table 1).

Patients perceived their own illness through the prism of its chronicity (chronic disease of own disease (IPQ 2) − M = 8.63). Slightly lower values were obtained for the components describing the impact of illness on a patient’s life (impact of disease on patient’s life (IPQ 1) − M = 7.08) and understanding of own illness (understanding own illness (IPQ 7) − M = 7.23) (Table 2).

The image of own illness as measured by the IPIS was dominated by destruction of physical sphere (destruction of physical sphere IPIS − M = 4.08), followed by pessimism (pessimism IPIS − M = 3.52) and destruction of mental sphere (destruction of mental sphere IPIS − M = 3.52) (Table 3).

The analysis shows that statistically significant correlations were observed in the study population between almost all components of the Brief-IPQ and self-assessed health status of the subjects (Table 4):

1. Impact of illness on the patient’s life (IPQ 1): *p*—0.001;
2. Chronicity of own illness (IPQ 2): *p*—0.001;
3. Controlling own illness (IPQ 3): *p*—0.001;
4. Confidence in the treatment of own illness (IPQ 4): *p*—0.01;
5. Perception of disease symptoms (IPQ 5): *p*—0.001;
6. Worrying about own illness (IPQ 6): *p*—0.001;
7. Negative effect of illness on emotional state (IPQ 8): *p*—0.001.

There were no statistical differences between genders in terms of perception of own illness measured by the Brief-IPQ scale. Marital status and education also did not affect the perception of own illness. Place of residence in the study population significantly affected the perception of own illness as measured by the Brief-IPQ. The differences are evident in terms of controlling own illness (*p*—0.00). Persons residing in the urban environment declared significantly greater confidence in controlling their own disease than patients living in rural areas. Overall, a low and medium correlation was shown between the results obtained.

The results of this study indicate that there were statistically significant correlations between the perception of own illness measured by the IPIS and self-assessed health status and duration of illness among the study population (Table 5). In relation to the self-assessed health status, the relations were as follows: loss of motivation for targeted activity IPIS: *p*—0.00; destruction of mental sphere IPIS: *p*—0.00; destruction of physical sphere IPIS: *p*—0.00; pessimism IPIS: *p*—0.00; and loss of control over the illness IPIS: *p*—0.00. With regard to the second variable, duration of illness, it correlates with the components: destruction of physical sphere IPIS: *p*—0.03; threat to the environment IPIS: *p*—0.02; and loss of control over the illness IPIS: *p*—0.02.

The results also show statistically significant gender differences in the perception of own illness measured with the IPIS. Men perceived their own illness to a significantly greater extent in terms of the destruction of mental sphere than women. Statistically significant differences were also observed, conditioned by the place of residence, in the perception of own illness measured with the IPIS. These differences concern the following component: loss of control over the illness (*p*—0.04). Residents of rural areas (M—3.28) were less convinced about the possibility of controlling their own disease than inhabitants of cities (M—2.90). Respondents’ education had a statistically significant effect on the perception of own illness measured by the IPIS in relation to the component: pessimism (*p*—0.04). Overall, there was a low correlation between the results obtained.

## 5. Discussion

The perception of own illness significantly affects the patient’s diagnostic and therapeutic processes. It is assumed to be the patient’s common-sense belief about their own illness, described in the cognitive and emotional concepts. The cognitive concept includes identification with the disease, its duration, control and treatment, consequence and consistency of the disease, while the emotional concept is closely related to the experienced emotions, namely, fear, anger and suffering [13].

Within the scope of the study subject, two standardized tools that analyze the perception of own illness were used, i.e., the Illness Perception Questionnaire Brief-IPQ and Imagination and Perception of Illness Scale IPIS. They allowed us to verify the aim of the study: how the elderly perceive their own illness.

For the study of the perception of own disease, the authors chose the respondents on the basis of a random selection. This limits the possibility of formulation based on the analyzed results of very general conclusions. The counterweight to this limitation, however, is the relatively large population of respondents, as well as the use of more than one research tool. Correspondence of the results obtained on the IPIS with the data collected using the Illness Perception Questionnaire Brief-IPQ should be an argument for their non-accidentality. Both questionnaires examine the perception of one’s own illness in as much detail as possible. A limitation to the above generalizations, however, is the simultaneous, and not prospective, nature of the research carried out. To some extent, it also limits the possibility of drawing conclusions about the influence of the disease situation on the nature of the relationship between its perception and health beliefs and the sense of meaning in life [8].

The nature of the results obtained in the course of the research carried out may be an important indication for medical personnel working with chronically ill persons. The image of the patient’s own disease is important for the functioning of the patient, making decisions and actions that are important in the aspect of his cooperation with the medical staff [8].

As a result of the conducted study, it should be noted that elderly people perceive their own illness through the prism of mental and physical destruction. However, respondents felt that the disease affected their physical sphere more than their mental sphere. It was also associated with a high level of motivation loss to undertake given activities. Similar results were obtained in the study by Król et al. (2016), who investigated the perception of own illness among people with multiple sclerosis projection-remission. Respondents perceived the disease similarly to our own study, namely, more often as a destruction of the physical sphere than the mental sphere. In addition, in both studies, analyses showed that the disease does not pose a threat to the environment [14].

Obtaining similar results of the research by the authors of this study and Król et al. (2016) indicates that over the years, the problem of perceiving one’s own illness has unfortunately not changed, regardless of the group under study. Statements that the respondents perceived their own disease through the prism of physical and mental destruction should be guidelines for medical staff to communicate with the patient as much as possible, talk about the disease and change their perspective.

Our own research showed a relationship between the destruction of mental sphere and the gender of respondents; men more often than women declared the above relationship, while Król et al. (2016) obtained the opposite relationship. In addition, in their analysis, statistical differences between age and illness perception were noted: people over 37 years of age perceived illness in a more pessimistic way than younger people and believed that they had less control over it [14]. In both studies, there were no statistically significant differences between illness perception and marital status of respondents. Sak et al. (2011) also observed that in a relationship among chronically ill people the destruction of physical sphere was more severe, but no statistical differences were observed between gender and the relationship in question [15]. A later study conducted by Sak (2013) found that men struggling with a chronic illness compared to women perceived their illness as threatening to the environment to a greater extent. Moreover, the respondents’ age correlated with the perception of their own illness through the prism of the destruction of the physical sphere [8]. The second of the Brief-IPQ questionnaires allowed us to draw conclusions on the perception of own illness among the elderly: illness significantly affects the lives of respondents. It is characterized by high chronicity, and seniors in a medium way control it, are convinced of the possibility of recovery and significantly feel the disease symptoms while understanding it; however, quite often they are worried about its presence and moderately feel the negative effects of disease on the emotional state.

A study using the aforementioned questionnaire was conducted by Saarti et al. (2016) among patients with heart disease receiving cardiac treatment in Beirut. The results obtained by them indicate different outcomes from the authors of this study regarding some issues. Cardiac patients assessed that the disease was unlikely to affect their daily life and they did not worry about its presence, and they were aware of the treatment and control options. The vast majority of respondents answered that the ailment did not negatively affect their emotional state [16].

Cardiac patients were also studied by Princip et al. (2018), with the difference that they were post-myocardial infarction patients, whose average age was 60.3 years, of Caucasian origin. The results of the study showed statistically significant differences between gender and control of own illness; men declared a higher control of illness than women. In addition, older patients had fewer problems with the disease than younger patients and assumed that the disease would last less time. Patients with higher levels of education reported longer disease duration [17]. The results of our study show statistically significant differences between controlling own illness and the respondents’ place of residence: subjects living in urban areas control their illness to a greater extent than those living in rural areas. This may be related to better accessibility to medical assistance, increased education and preventive health care. In addition, the elderly living in cities benefit from various meetings held at the Universities of the Third Age, seniors’ clubs and housing estate clubs, which focus on the prevention of diseases that affect them. With such meetings, the awareness of seniors increases. In another study, seniors struggling with heart failure described their disease as a chronic condition with little variability but negatively affecting their lives and emotional state; nevertheless, they understood and controlled it through self-management and treatment [18].

Zielazny (2016), among others, assessed disease perception among people with chronic obstructive pulmonary disease in his study by dividing them into two subgroups: group I—patients with first and second degree of spirometric classification of COPD; group II—patients with third and fourth degree. Comparing these two groups, he determined that subjects in group II experienced a greater impact of COPD on their lives than those in group I, and considered the disease to be more chronic, incurable and life-long. In addition, they were more worried about its presence, did not fully understand its presence and it significantly affected their emotional state. The author of this study also noted statistical differences related to the perception of COPD and the place of residence of the subjects. In group I, as the number of inhabitants in the place of residence increased, the perception of illness with respect to the category of illness duration decreased, and those living in large cities were less likely to describe the illness as incurable [19]. Turkish patients with rheumatoid arthritis in the perception of their illness attached great importance to its chronicity and existential consequences. Moreover, they severely felt its negative effects in relation to the emotional sphere [20].

Similar results were obtained by Cordingley et al. (2014) also among people with RA, only hospitalized in the UK. The analysis determined that RA has a significant impact on patients’ lives, has multiple consequences and the vast majority of those surveyed experienced severe disease symptoms. Patients also reported low levels of understanding the disease [21]. Patients with type II diabetes also had a different perception of their illness than the study population. They believed themselves to be able definitively to control their disease and it was only up to them how the disease progressed [22]. In contrast, people with the disease living in Tripoli, Libya, similarly to the study population, described the disease as highly chronic but controllable and treatable. Regarding the impact on emotional state, respondents described it as having a moderate negative impact on this area of life [23].

Research among patients with RA, type 1 diabetes and ischemic heart disease was also conducted by Ziarko (2014). His study found that “in the subjective experience of illness, RA patients experience the most symptoms and type 1 diabetes patients the least (…) Diabetic patients, compared to ischemic heart disease and RA patients, are more confident that they can influence their own health, that their personal activity will translate into health, and are able to influence the course of treatment, which is likely to be successful. In contrast, RA patients are characteristically unconvinced that they can control their own health and actively engage in the treatment process [24].”

Interesting results of the study were also obtained by Alharbi et al. (2017), who analyzed the perception of illness among patients with end-stage renal disease undergoing hemodialysis and peritoneal dialysis. The majority of respondents perceived disease progression over time. Similar to the surveyed seniors of the present study, they believed that the disease significantly affected their daily lives while understanding it. However, based on their high scores on personal disease control and treatment, the respondents believed that they had the ability to control their disease symptoms, and that their illnesses could be treated. However, they expressed unstable emotional responses. The authors further divided the patients into two subgroups: those undergoing hemodialysis (HD) and peritoneal dialysis (PD). The HD patients compared to the PD patients described their disease as more chronic, while those on PD were significantly more confident in controlling their disease [25].

An analysis of own studies identified the relationship between perception of own illness and the subjective assessment of the health status of the subjects. The elderly describing their health status as good perceived their illness as less troublesome. Galhenage et al. (2016) studied psychological morbidity and illness perception among TB patients in Sri Lanka. Their results show that illness-related depression and anxiety are common among the above patients. Moreover, these patients have significantly higher scores on the Brief-IPQ than patients with only TB [26].

The research carried out by the authors and other authors, discussed above, shows that the perception of one’s own illness may be different depending on the age of the studied group, its place of residence and the type of illness with which the respondents are accompanied. Our own research, in comparison with other analyses, did not indicate any relationship between the respondents’ gender and age, and showed a relationship between the self-assessment of health conditions. The authors of the study wanted to indicate how the health condition influences the perception of own disease among the elderly. It was possible thanks to the use of the IPIS. However, this does not change the fact that more research is needed on the perception of own disease among older people, carried out on a larger scale.

## 6. Conclusions

The study on the perception of own illness especially in the elderly group is particularly important. According to the study, seniors perceive their own illness through the prism of mental and physical destruction. In the case of mental destruction, in addition to the disease burden, low awareness of the spectrum of disease, lack of support in loved ones and lack of understanding on the part of medical staff may influence this state.

It is advisable, especially for this social group, to have a positive perception of their own illness, to understand it and to be able to function with it to the best of their ability. A negative perception of illness, in addition to its subsequent health consequences (depression, mental breakdowns, anxiety) may lead to problems in the functioning of health care, as a result of a lack of trust in medical staff and the search for further possible therapeutic processes. Medical staff and the immediate family should participate in shaping a positive perception of illness among seniors.

## Figures and Tables

**Table 1:** Sociodemographic data.

Sociodemographic Data		
	%	N
Age		
Women	51.82	157
Men	48.18	146
Place of residence		
City	67.32	204
Out of town	32.67	99
Marital status		
Married	49.17	149
Single	18.15	55
Cohabitation	15.18	46
Widowed	8.92	27
Divorced	8.58	26
Education level		
Secondary education	41.25	125
Vocational education	28.38	86
Higher education	27.07	82
Primary education	3.30	10

**Table 2:** Descriptive statistics regarding the perception of own illness as measured by the Brief-IPQ.

Dependent Variables	Descriptive Statistics							
	N	M	Me	Min.	Max.	(Q1)	(Q3)	SD
	Study Population							
Impact of illness on the patient’s life (IPQ 1)	303	7.08	8.00	0	10.00	6.00	9.00	2.55
Chronicity of own illness (IPQ 2)	303	8.63	10.00	0	10.00	8.00	10.00	2.44
Controlling own illness (IPQ 3)	303	5.22	5.00	0	10.00	3.00	8.00	2.90
Confidence in the treatment of own illness (IPQ 4)	303	5.70	6.00	0	10.00	3.00	8.00	2.96
Perception of disease symptoms (IPQ 5)	303	6.73	7.00	0	10.00	5.00	9.00	2.57
Worrying about own illness (IPQ 6)	303	6.11	7.00	0	10.00	4.00	8.00	3.00
Understanding own illness (IPQ 7)	303	7.23	8.00	0	10.00	5.00	10.00	2.57
Negative effect of illness on emotional state (IPQ 8)	303	4.51	4.00	0	10.00	3.00	5.00	2.25

N—study population, M—arithmetic mean, Me—median, Min.—minimum, Max.—maximum, Q1—first quartiles, Q3—third quartiles, SD—deviation standard.

**Table 3:** Descriptive statistics regarding the perception of own illness as measured by the IPIS.

Dependent Variables	Descriptive Statistics by Study and Control Group							
	N	M	Me	Min.	Max.	(Q1)	(Q3)	SD
	Study Population							
Loss of motivation for targeted activity IPIS	303	3.50	3.38	0.00	6.00	2.75	4.50	1.32
Destruction of mental sphere IPIS	303	3.52	3.50	0.00	6.00	2.67	4.67	1.36
Destruction of physical sphere IPIS	303	4.08	4.25	0.38	6.00	3.38	5.00	1.14
Pessimism IPIS	303	3.52	3.50	0.00	6.00	2.75	4.50	1.44
Threat to the environment IPIS	303	0.62	0.00	0.00	5.00	0.00	1.00	0.98
Loss of control over the illness IPIS	303	3.02	3.00	0.00	6.00	2.00	4.00	1.49

N—study population, M—arithmetic mean, Me—median, Min.—minimum, Max.—maximum, Q1—first quartiles, Q3—third quartiles, SD—deviation standard.

**Table 4:** Perception of own illness as measured by the Brief-IPQ vs. age, self-assessed health status and duration of illness.

Variables		Age		Self-Assessed Health Status		Duration of Illness	
	N Significant	R Spearman	*p*	R Spearman	*p*	R Spearman	*p*
	Study Group						
Impact of illness on the patient’s life (IPQ 1)	303	0.06	0.28	−0.41	0.001 *	−0.01	0.80
Chronicity of own illness (IPQ 2)	303	0.03	0.57	−0.17	0.001 *	0.09	0.13
Controlling own illness (IPQ 3)	303	0.05	0.40	0.25	0.001 *	−0.03	0.55
Confidence in the treatment of own illness (IPQ 4)	303	0.04	0.53	0.16	0.01 *	−0.06	0.33
Perception of disease symptoms (IPQ 5)	303	0.03	0.63	−0.41	0.001 *	−0.05	0.42
Worrying about own illness (IPQ 6)	303	0.05	0.43	−0.39	0.001 *	−0.01	0.89
Understanding own illness (IPQ 7)	303	0.00	0.98	0.09	0.13	0.02	0.74
Negative effect of illness on emotional state (IPQ 8)	303	0.08	0.17	−0.27	0.001 *	0.01	0.92

N—study population, R Spearman—Spearman’s rank correlation coefficient—rho, *p*—significance interval statistical, *—*p* > 0.05.

**Table 5:** Perception of own illness as measured by IPIS vs. age, self-assessed health status and duration of illness of the respondents.

		Age		Self-Assessed Health Status		Duration of Illness	
Dependent Variables	N Significant	R Spearman	*p*	R Spearman	*P*	R Spearman	*p*
	Study Group						
Loss of motivation for targeted activity IPIS	303	−0.03	0.58	−0.38	0.001 *	0.09	0.13
Destruction of mental sphere IPIS	303	−0.00	0.98	−0.37	0.001 *	0.12	0.03 *
Destruction of physical sphere IPIS	303	0.02	0.64	−0.40	0.001 *	0.07	0.22
Pessimism IPIS	303	−0.03	0.53	−0.40	0.001 *	0.08	0.15
Threat to the environment IPIS	303	−0.03	0.59	0.02	0.77	−0.13	0.02 *
Loss of control over the illness IPIS	303	−0.05	0.36	−0.38	0.001 *	0.14	0.02 *

N—study population, R Spearman—Spearman’s rank correlation coefficient—rho, *p*—significance interval statistical, *—*p* > 0.05.

## Data Availability

All information about the data is available from the authors. Original data will be provided by the authors if necessary.
